# Exploring the factors affecting musculoskeletal disorders risk among hospital nurses

**DOI:** 10.1371/journal.pone.0231319

**Published:** 2020-04-16

**Authors:** Shu Chuan Lin, Li Li Lin, Chih Ju Liu, Chun Kai Fang, Mei Hsiang Lin

**Affiliations:** 1 Department of Nursing, Mackay Memorial Hospital, Taipei,Taiwan, Republic of China; 2 Mackay Memorial Hospital, Taipei, Taiwan, Republic of China; 3 Department of Nursing, National Taipei University of Nursing and Health Sciences, Taipei, Taiwan, Republic of China; Instituto Federal Goiano, BRAZIL

## Abstract

**Background:**

Musculoskeletal disorder (MSD) is currently recognized as one of the most common occupational injuries for which nursing personnel in the medical service industry have been identified as a high-risk group. In this study, we explore the prevalence of MSD in various body parts as well as their risk factors among hospital nurses.

**Methods:**

A cross-sectional descriptive design with stratified cluster sampling was used to collect data from 1,803 nurses. The survey included a demographic questionnaire, and Nordic Musculoskeletal Questionnaire.

**Results:**

The results showed that the greatest prevalence of MSD symptoms by body regions were in the right shoulder (85.8%), the left shoulder (80.9%), the neck (62.4%), the right wrist (62.2%) and the lower back (60.4%). Risk factors for shoulder discomfort includes department type, exercise habits, and age (*p* < .05). Risk factors for neck discomfort includes seniority in the current unit, “job title, and “history of MDS (*p* < .05). Risk factors for upper back discomfort includes age and seniority in the current unit (*p* < .05). Risk factors for lower back discomfort including seniority in the current unit, department type, and number of days worked per week (*p* < .05).

**Conclusions:**

The results of this study can serve as a reference for nursing administration managers and decision-makers for reducing musculoskeletal discomfort among nurses and thereby achieving superior quality in clinical care.

## Introduction

Musculoskeletal disorder (MSD) is defined as connective tissue or musculoskeletal disease that causes muscle pain or injury due to sudden or continuous contact, repeated exercise, force, vibration, or incorrect posture movements [1,2]. MSD has been recognized as one of the most common occupational injuries, and nurses in the medical service industry have been identified as a high-risk group for such injuries [3]. The worldwide epidemiological studies have revealed a high incidence and prevalence of low back musculoskeletal complaints among nurses [4]. Furthermore, work-related musculoskeletal disorders (WMSDs) are among the leading causes of occupational diseases among healthcare professionals worldwide [5–7]. In a clinical setting, nursing personnel often must move, pull, or push patients to and from their beds or other locations, and such activities involve excessive bending or turning of the body [8,9]. In addition, awareness of physical discomfort can affect an individual’s daily life, and when severe, the individual may need to take leave from work or seek medical help. Physical discomfort can even reduce an individual’s capacity to work or cause disabilities, which in turn causes a burden to employers and society [10]. Nursing is an occupation with a high prevalence of MSDs, which can easily lead to reductions in the nursing workforce and affect negatively nursing [11–13].

The prevalence of work-related MSD among nurses varies between studies. Salama and Eleshenamie [14] studied the risk factors of MSD and coping strategies among 300 nurses in outpatient departments and intensive care units. They found that a high proportion of nurses reported MSD (99.0%) during the last year. Tinubu, Mbada, Oyeyemi, and Fabunmi [15] studied Nigerian nurses and found that those with more than 20 years of clinical work experience reported greater discomfort from work-related MSDs than did nurses with 11–20 years of work experience. A multicenter cross-sectional study by Clari et al. [6] showed that 48.3% of operating room nurses reported one or more episodes of upper limb pain. Moreover, a systematic review and meta-analysis by Saberipour, Ghanbar, Zarea, Gheibizadeh, Zahedian [7] showed that the prevalence of MSD and lower back pain in nurses were 0.84 (95% CI: 0.83–0.95) and 0.60 (95% CI: 0.60–0.61) respectively. Kalkim, Midilli, and Dogru [16] conducted a survey on 498 nurses with MSDs. Their results indicated that the body locations with the highest prevalence rates were the lower back (78.5%), back (74.9%), knee joint (63.1%), neck (61.2%), and shoulder (59.6%).

Numerous studies have shown that nurses have one of the highest prevalence rates for MSD, which is a factor that contributes to reductions in the nursing workforce and negatively affects the quality of care [3]. The highest prevalence rate for MSD among Taiwanese nurses is also suggested by various studies, such as the Survey of Perceptions of Safety and Health in the Work Environment. From August 2016 to December 2017, in Taiwan, 65.16% of employees reported feeling physical discomfort in the previous year. Of the body parts affected by discomfort, the shoulder (41.31%), neck (32.25%), and lower back or waist (31.03%) accounted for the highest proportion [17]. Another study conducted in Taiwan on the causes of MSD among nurses, the average prevalence rate of MSD among nurses staffed at a medical center nurses is 34.2%. These nurses had prevalence rates of 62.6% for shoulder discomfort, 63.5% for neck discomfort, and 59.3% for lower back discomfort [18]. MSDs consisted of sprains, strains, lacerations, pain in the back or other locations, carpal tunnel syndrome, muscle systems and connective tissue diseases caused by bending of the waist, grasping, twisting, excessive use of force, or repetitive actions and are the most common occupational injury among professional care workers [1]. Other than acute trauma, the causes of most cases of MSD are repetitive actions, excessive load-bearing and the slow development of poor posture over an extended period of time [3]. Cheng et al. [19] administered the Nordic Musculoskeletal Questionnaire to 470 nurses and discovered that the lower back (77.2%), neck (64.2%), and shoulder (58.7%) were the locations on the body with the highest prevalence rates of MSD. A study conducted by Chen et al. [18] indicated that correlations exist between the workload and work satisfaction of nurses and their degree of musculoskeletal pain.

The Bureau of Labor Insurance [20] indicated that MSD was the number one cause of occupational injuries among individuals receiving compensation through labor insurance. In 2006, MSD accounted for 50.3% of occupational injuries; by 2014, this proportion had reached 62.5%. Chen et al. [18] investigated musculoskeletal discomfort among nurses at 793 medical centers, and their results indicated that 63.5% had neck pain, 62.6% had shoulder pain, and 59.3% had lower back pain.

Several studies have indicated that basic attributes of an individual are factors that affect the risk of developing MSD. The origin of MSDs is complex and multi-factorial. Body Mass Index (BMI) is associated with MSDs in various regions of the body [21]. A high BMI (overweight and obesity) was moderately associated with an increased prevalence of musculoskeletal symptoms [22]. Also, according to Chen et al. [23] indicated that influential factors that contribute to MSD include age, work seniority, work content, working hours, number of hours worked per week, amount of time standing or walking during work, stress levels from work, and exercise habits. Ko et al. [24] discovered a significant correlation between turning or moving patients and pain and discomfort in the lower back. Bazazan et al. [25] investigated the association of MSD and workload with work schedule and job satisfaction among emergency care nurses, and found a significant negative correlation between MSDs prevalence in all body regions, with the exception of the hips/thighs, and degree job satisfaction. Moreover, Hoogendoorn et al. [26] postulated that improving job satisfaction and social support at work may contribute to the prevention of sickness as well as being absent due to lower back pain. Chen et al. [18] reported that more than 50% of nursing staff in their study experienced discomfort with their neck, lower back, and shoulder, and that those with a lower quality of life were 1.6–3.0 times more likely to experience musculoskeletal pain than were nurses with a high quality of life. Nurses with low levels of work satisfaction were 1.7–2.0 times more likely to suffer from MSD than were nurses with high levels of work satisfaction. To the best of our knowledge, understanding the prevalence and factors associated with MSDs among nurses is important for health policy administrators and health-care workers to curtail the existence of the problem. Thus, the distribution of MSD among nurses must be determined. In this study, the Nordic Musculoskeletal Questionnaire was used to explore the prevalence of MSD in different body locations and their predictors in nurses. Results from this study can serve as a reference for nursing administration managers and decision-makers in reducing musculoskeletal discomfort among nurses and thereby achieving improved nursing quality and performance.

## Methods

### Design, subjects and setting

A cross-sectional design which used a self-administered questionnaire survey was employed. All participants were recruited from a single northern Taiwan medical center. It has a total of 2,089 beds and for the purpose of this study were divided into internal medicine, surgical department, obstetrics & gynecology, intensive care unit, pediatric department, and others (burn unit, outpatient department, and casualty unit). A total of 2,161 registered nurses in that institution covered internal medicine, surgery department, obstetrics & genecology, intensive care unit, and pediatric department. They worked 40 hours a week and sometimes worked overtime. Taiwan’s clinical nursing staff is divided into N1, N2, N3 and N4 clinic al ladder levels [27]: N1 is responsible for basic care, N2 is responsible for intensive care, N3 is responsible for education and overall care, and N4 is responsible for research and professional care. A convenient sampling was utilized to collect the data. The eligibility criteria for participants were as follows: (1) registered nurses who had worked on the hospital for at least 4 hours per day or 20 hours per week, (2) above 20 years of age, and (3) were willing to participate in the study. Sample size was calculated according to the World Health Organization Guideline [28] and based on a previous study by Hou and Shiao [29]. There were 2,242 participants recruited who met the inclusive criteria recruited. Observed individuals who have any missing data will be excluded from the study and will not be included in the sample for analysis. Three hundred and eighty-eight participants who did not complete the questionnaire were excluded from this study. In total 1,803 participants took part in this study, with a response rate of 82.69%.

#### Study period

The study period was from November 2011 to January 2012.

#### Data collection

All participants were asked to fill out the questionnaire in their own time and then to place it into a box that was located outside their worksite within 7 days. Participants were reassured that their participation was entirely voluntary. The demographic characteristics questionnaire and the Nordic Musculoskeletal Questionnaire lasted for 10 to 20 minutes.

### Instruments

#### Demographic characteristics questionnaire

Gender, job title (administrative supervisor, full-time nurses, part-time nurses, other), department type (internal medicine, surgical department, obstetrics & gynecology, pediatric department, intensive care unit, other), work mode (fixed, three-shift rotation, (8 hours/day), other), daily work rest time, history of MSD (yes or no), age, exercise habits, body weight, work seniority, number of hours worked per day, number of days worked per week, and BMI. Each group’s descriptive statistics are expressed in terms of number of people and only reflect current symptoms.

#### Nordic Musculoskeletal Questionnaire

This study used the Nordic Musculoskeletal Questionnaire [30] to investigate the common types of MSD seen in the work field and the location of the injury. The interviewees were asked to select from nine body locations (shoulder, neck, lower back, upper back, elbow joint, hand or wrist, hip joint, knee joint, and ankle joint). The questions include whether they have felt discomfort in the past year, whether they have felt discomfort in the past week, and whether the discomfort affected their life or ability to work (a “yes” response was given a score of 1 and a “no” response was given a score of 0). The highest discomfort score for each location was three points, and the lowest was 0 points. Because the score scope was limited, the scores for discomfort at each location were further categorized as “yes” (0) and “no” (1–3 points = 1). This questionnaire was designed as a standardized questionnaire and can be used to further investigate the symptoms of a specific location (see S1 File). The questionnaire can identify differences in symptoms caused by different work stations, thereby enabling MSD problems to be more clearly defined. Numerous studies have demonstrated that this questionnaire has adequate internal consistency, reliability, and validity [30,31].

#### Ethical considerations

This study was approved by the Ethical Review Committee of the Mackay Memorial Hospital Institutional Review Board (approval No. 11MMHIS125). The objectives of the study and the questionnaire were explained to all of the participants and they were assured of their anonymity as well as confidentiality of their responses. All the participants provided a signed informed consent to participate.

### Data analysis

Data was analyzed using the IBM SPSS software statistical version 23.0 for Windows (IBM, Armonk, NY, USA). Prevalence of musculoskeletal discomfort was analyzed using descriptive statistics. Chi-square tests were used to examine variations in musculoskeletal discomfort prevalence between difference participants’ demographics. Logistic regression was performed to determine risk factors related to musculoskeletal discomfort. Odds ratios (OR) were calculated to determine the contribution of each risk factor for shoulder and neck/back, and a *p* value of less than .05 was considered statistically significant. Each group’s statistics are expressed in terms of number of people those who had musculoskeletal discomfort.

## Results

Table 1 shows that the average age of participants in this study was 36.63 ± 11.24, and 99.06% were women. Overall, 76.59% rarely exercise, 87.52% had no history of MSD, and 68.61% were full-time nurses. Most of the participants were nurses in the internal medicine department. Of the participants, 60.29% worked a three-shift rotation, the average number of years working was 11.61 ± 9.33, and the average number of hours worked per day was 8.90 ± 1.51. Of the nine major musculoskeletal locations where discomfort was felt, the locations most commonly reported were the right shoulder at 85.8%, followed by the left shoulder at 80.9% and the neck at 62.4%. About half of the nurses were normal weight (54.1%). (see S2 File).

**Table 1:** Characteristics of participant and demographics. (N = 1803).

Variables		n	%	Mean (SD)
Gender	Female	1786	99.06	
	Male	17	0.94	
job title	administrative supervisor	140	7.76	
	full-time nurses	1237	68.61	
	part-time nurses	147	8.15	
	Other	279	15.47	
department type	internal medicine	480	26.6	
	surgical department	247	13.7	
	obstetrics & gynecology	108	6.0	
	pediatric department	212	11.8	
	intensive care unit	287	15.9	
	Other	469	26.0	
work mode	Fixed	567	31.45	
	three-shift rotation (8 hours/day)	1087	60.29	
	Other	149	8.26	
daily work rest time	no	1075	59.62	
	Yes	728	40.38	
exercise habits	Rarely	1381	76.59	
	once a week	238	13.20	
	twice a week	96	5.32	
	three times a week	88	4.88	
history of MSD	No	1578	87.52	
	Yes	225	12.48	
^#^ Shoulder, neck, back and upper limb discomfort	Neck	797	62.4	
	left shoulder	757	80.9	
	right shoulder	804	85.8	
	upper back	414	32.9	
	lower back	764	60.4	
	left elbow joint	192	44.3	
	right elbow joint	235	53.3	
	left wrist	246	38.2	
	right wrist	389	62.2	
^#^ Lower limb discomfort	left knee joint	289	60.1	
	right knee joint	292	59.7	
	left ankle joint	228	47.0	
	right ankle joint	212	47.3	
	left hip joint	195	40.8	
	right hip joint	195	41.7	
^&^**BMI**	< 18.5	221	12.3	
	18.5≦BMI<24	974	54.0	
	24≦BMI<27	226	12.5	
	27≦BMI<30	102	5.7	
	BMI≧30	55	3.1	
Age				36.63±11.24
body weight				55.78±9.78
work seniority				11.61±9.33
number of hours worked per day (hour)				8.90±1.51
number of days worked per week (day)				5.07±1.09

^#^ Each group’s descriptive statistics are expressed in terms of number of people those who had musculoskeletal discomfort.

^&^Because of missing or incomplete data, the total of all variables might not equal to 1803.

### Association between participants’ demographics, neck, shoulder, and back discomfort

Neck, shoulder and back discomfort referred to discomfort in the neck, left shoulder, right shoulder, or lower back. A chi-square test was performed to determine the association between participants’ demographics, neck, shoulder, and back discomfort. The study results revealed the following: (1) age, work seniority, seniority in the current unit, job title, number of hours worked per day, hours standing per day, number of days worked per week, and history of MDS were significantly associated with those who experienced neck discomfort (χ^2^ = 18.41, 21,92, 32.93, 23.01, 32.48, *p* < .001, χ^2^ = 12.34, *p* = .015, χ^2^ = 17.02, *p* < .01, χ^2^ = 7.68, *p* = .006); (2) age, work seniority, job title, department type, number of hours worked per day, and exercise habits were significantly associated with those who experienced left shoulder discomfort (χ^2^ = 9.75, *p* = .021, χ^2^ = 11.56, *p* = .009, χ^2^ = 10.23, *p* = .017, χ^2^ = 18.86, *p* = .004, χ^2^ = 9.32, *p* = .025, χ^2^ = 11.57, *p* = .009); (3) age and number of hours worked per day were significantly associated with those who right shoulder discomfort (χ^2^ = 9.90, *p* = .019, χ^2^ = 9.14, *p* = .027); (4) age, work seniority, seniority in the current unit, department type, and work mode were significantly associated with those who upper back discomfort (χ^2^ = 22.64, 17.07, *p* < .001, χ^2^ = 13.57, *p* = .035, χ^2^ = 11.92, *p* = .008); (5) age, work seniority, seniority in the current unit, department type, number of hours worked per day, number of days worked per week, BMI, and BW were significantly associated with those who lower back discomfort (χ^2^ = 8.82, *p* = .032, χ^2^ = 14.82, *p* = .002, χ^2^ = 17.85, *p* = .001, χ^2^ = 16.07, *p* = .013, χ^2^ = 14.33, *p* = .002, χ^2^ = 17.42, *p* < .001, χ^2^ = 9.84, *p* = .043, χ^2^ = 12.13, *p* = .016) (Table 2).

**Table 2:** Shoulder, neck, back, and upper limb muscle discomfort in nurses across different demographic characteristics^#^.

Variables	neck	left shoulder	right shoulder	upper back	lower back	left elbow	right elbow	left wrist	right wrist	^§^multiple sites
	n = 797 62.4%	n = 757 80.9%	n = 804 85.8%	n = 414 32.9%	n = 764 60.4%	n = 192 44.3%	n = 235 53.3%	n = 246 38.2%	n = 389 62.2%	n = 1555 86.2%
**age (years)**										
< 30	53 (44.5)	62 (79.5)	58 (75.3)	20 (16.8)	57 (47.9)	8 (36.4)	9 (34.6)	11 (25.0)	13 (32.5)	516(83.8)
30 to< 40	331 (63.7)	313 (84.6)	328 (88.4)	160 (31.1)	318 (61.4)	50 (37.0)	62 (46.6)	77 (35.0)	124 (58.5)	462(88.0)
40 to<50	210 (66.0)	196 (82.0)	207 (87.0)	110 (34.9)	197 (62.7)	49 (44.5)	58 (51.8)	73 (38.0)	130 (71.0)	365(90.8)
≧50	203 (63.2)	186 (74.7)	211 (84.1)	124 (40.1)	192 (61.3)	85 (51.2)	106 (62.4)	85 (45.2)	122 (65.6)	165(85.1)
χ^2^	18.41	9.75	9.90	22.64	8.82	6.66	11.73	8.12	23.29	11.74
*p*	< .001	.021	.019	< .001	.032	.084	.008	.044	< .001	.008
**work seniority(years)**										
< 10	181 (52.5)	189 (80.8)	192 (83.1)	82 (24.0)	188 (54.5)	32 (36.8)	35 (38.9)	46 (33.3)	57 (45.2)	778(83.9)
10–19	318 (65.4)	295 (83.3)	314 (87.5)	172 (35.9)	298 (62.1)	72 (46.8)	82 (52.2)	88 (37.3)	140 (61.4)	382(89.9)
20–29	199 (69.1)	190 (83.0)	197 (87.6)	105 (37.0)	194 (68.1)	61 (49.6)	75 (60.5)	71 (39.2)	130 (73.9)	261(88.8)
≧30	99 (62.3)	83 (69.7)	101 (82.8)	55 (35.9)	84 (54.5)	27 (39.1)	43 (61.4)	41 (46.1)	62 (68.1)	80(87.9)
χ^2^	21.92	11.56	3.66	17.07	14.82	4.51	12.01	3.88	27.10	10.81
*p*	< .001	.009	.300	< .001	.002	.211	.007	.274	< .001	.013
**seniority in the current unit (years)**										
<1	76 (43.7)	75 (77.3)	77 (80.2)	34 (19.7)	81 (46.6)	13 (36.1)	12 (31.6)	16 (26.2)	23 (41.8)	177(76.0)
1 to< 3	130 (61.6)	134 (82.2)	139 (86.3)	58 (27.8)	133 (63.0)	23 (34.8)	32 (48.5)	35 (38.0)	47 (54.0)	243(86.8)
3 to< 5	113 (62.8)	98 (81.7)	110 (85.9)	58 (32.4)	110 (61.8)	24 (46.2)	24 (46.2)	27 (30.0)	51 (57.3)	229(89.8)
5 to< 10	162 (65.6)	153 (82.7)	163 (88.6)	81 (33.6)	145 (59.7)	38 (45.2)	49 (55.7)	53 (41.7)	78 (62.4)	290(87.3)
≧10	316 (67.8)	297 (80.1)	315 (85.6)	183 (40.1)	295 (64.4)	94 (48.2)	118 (59.9)	115 (42.0)	190 (71.7)	616(87.6)
χ^2^	32.93	1.59	3.69	27.13	17.85	4.67	12.53	8.59	23.33	25.01
*p*	< .001	.811	.450	< .001	.001	.322	.014	.072	< .001	< .001
**job title**										
administrative supervisor	51 (81.0)	40 (78.4)	49 (90.7)	26 (41.3)	38 (60.3)	10 (34.5)	16 (57.1)	10 (27.8)	20 (55.6)	129(92.1)
full-time nurses	615 (64.0)	590 (82.9)	611 (86.7)	302 (31.9)	580 (61.1)	124 (43.2)	151 (51.7)	175 (37.5)	290 (65.2)	1063(85.9)
part-time nurses	41 (50.6)	40 (80.0)	42 (82.4)	21 (26.3)	48 (59.3)	10 (47.6)	11 (50.0)	11 (31.4)	20 (57.1)	126(85.7)
Other	90 (52.0)	87 (70.7)	102 (80.3)	65 (38.9)	98 (57.6)	48 (50.0)	57 (57.6)	50 (47.2)	59 (56.2)	185(86.4)
χ^2^	23.01	10.23	5.15	6.81	0.75	2.63	1.29	6.05	4.31	4.22
*p*	< .001	.017	.161	.078	.861	.452	.733	.109	.230	.238
**department type**										
internal medicine	202 (61.4)	201 (81.7)	207 (83.1)	101 (31.4)	210 (64.4)	43 (39.4)	49 (45.0)	51 (32.3)	80 (54.8)	392(88.7)
surgical department	172 (64.9)	174 (88.3)	171 (87.2)	95 (36.3)	171 (64.5)	48 (48.0)	61 (58.1)	64 (44.8)	95 (69.3)	219(88.7)
obstetrics & Gynecology	58 (64.4)	56 (81.2)	61 (89.7)	35 (39.8)	47 (52.8)	12 (44.4)	16 (55.2)	15 (34.9)	30 (71.4)	94(87.0)
intensive care unit	113 (57.7)	95 (71.4)	107 (82.3)	49 (25.3)	107 (55.4)	25 (41.0)	27 (46.6)	35 (36.8)	46 (51.1)	178(84.0)
pediatric department	110 (62.1)	102 (82.9)	112 (88.2)	56 (32.4)	89 (51.4)	27 (60.0)	28 (62.2)	42 (45.2)	61 (66.3)	238(82.9)
Other more than 2 department	120 (64.2)	113 (78.5)	128 (89.5)	71 (38.4)	121 (65.4)	35 (42.7)	52 (60.5)	32 (32.3)	69 (68.3)	402(87.2)
χ^2^	3.22	18.86	8.45	13.57	16.07	8.22	11.83	9.93	14.89	7.36
*p*	.780	.004	.207	.035	.013	.222	.066	.128	.021	.195
**work mode**										
Fixed	213 (62.6)	194 (75.5)	221 (84.7)	118 (35.1)	197 (59.0)	66 (45.8)	81 (55.5)	66 (35.5)	116 (63.0)	506(89.2)
three-shift rotation (8 hours/day)	540 (61.7)	520 (83.1)	538 (86.2)	264 (30.7)	526 (60.6)	114 (43.3)	135 (50.6)	158 (38.3)	238 (60.4)	924(85.0)
Other	40 (69.0)	39 (81.3)	41 (85.4)	29 (50.0)	37 (64.9)	11 (50.0)	16 (66.7)	21 (51.2)	32 (84.2)	22(84.6)
More than two work mode	4 (80.0)	4 (80.0)	4 (100.0)	3 (60.0)	4 (80.0)	1 (25.0)	3 (75.0)	1 (20.0)	3 (60.0)	57(89.1)
χ^2^	1.91	6.78	1.03	11.92	1.58	1.13	3.56	4.23	8.42	6.17
*p*	.592	.079	.794	.008	.663	.771	.313	.238	.038	.103
**number of hours worked per day (hour)**										
≦7	7 (70.0)	9 (90.0)	7 (77.8)	3 (30.0)	5 (50.0)	2 (66.7)	1 (33.3)	1 (33.3)	0 (0.0)	84(84.8)
8	257 (52.6)	247 (76.0)	269 (81.5)	145 (30.5)	261 (54.4)	86 (48.3)	98 (54.1)	98 (39.5)	139 (58.4)	586(84.7)
9 to 11	475 (68.3)	439 (82.7)	464 (87.9)	234 (34.0)	438 (63.6)	86 (38.4)	117 (50.9)	124 (35.8)	221 (65.0)	758(88.7)
≧12	58 (69.0)	62 (88.6)	64 (91.4)	32 (37.6)	60 (70.6)	18 (64.3)	19 (70.4)	23 (48.9)	29 (70.7)	127(80.9)
χ^2^	32.48	9.32	9.14	2.50	14.33	9.47	4.24	3.32	7.14	9.56
*p*	< .001	.025	.027	.475	.002	.024	.237	.344	.068	.023
**standing (hour/ day)**										
1–4	180 (64.3)	165 (78.6)	179 (84.8)	91 (32.7)	159 (56.8)	43 (41.0)	50 (47.6)	40 (27.4)	81 (56.3)	391(88.1)
5	117 (67.2)	105 (83.3)	118 (89.4)	49 (28.8)	101 (58.7)	18 (38.3)	22 (48.9)	28 (34.1)	49 (62.0)	199(89.6)
6–7	337 (61.6)	313 (79.8)	326 (84.0)	179 (33.1)	326 (60.5)	84 (44.7)	103 (54.2)	118 (42.8)	167 (62.5)	598(86.2)
8	91 (52.3)	95 (77.9)	101 (83.5)	51 (30.4)	104 (61.2)	22 (42.3)	32 (54.2)	34 (40.5)	53 (65.4)	193(83.5)
≧9	72 (69.9)	79 (91.9)	80 (94.1)	44 (43.1)	74 (71.8)	25 (61.0)	28 (66.7)	26 (46.4)	39 (78.0)	174(82.1)
χ^2^	12.34	8.90	7.94	6.63	7.42	5.88	4.81	12.00	7.84	7.91
*p*	.015	.064	.094	.157	.115	.209	.307	.017	.098	.095
**number of days worked per week (day)**										
1–3	47 (65.3)	43 (82.7)	51 (92.7)	25 (35.2)	49 (69.0)	10 (47.6)	14 (63.6)	12 (36.4)	25 (71.4)	99(89.2)
4	38 (45.8)	41 (80.4)	42 (80.8)	20 (24.7)	38 (46.3)	10 (47.6)	12 (52.2)	12 (37.5)	16 (48.5)	111(84.1)
5	416 (60.5)	397 (79.2)	417 (84.1)	212 (31.4)	389 (57.5)	92 (43.4)	109 (50.7)	131 (40.1)	188 (60.3)	751(84.9)
6–7	296 (68.0)	276 (83.1)	294 (88.0)	157 (36.5)	288 (66.2)	80 (44.7)	100 (55.2)	91 (36.1)	160 (66.4)	54(90.3)
χ^2^	17.02	2.08	5.82	5.91	17.42	0.27	1.82	1.00	6.19	10.78
*p*	< .001	.557	.121	.116	< .001	.966	.611	.801	.103	.013
**exercise habits**										
Rarely	623 (62.5)	602 (82.6)	627 (85.7)	314 (32.0)	597 (60.7)	127 (39.4)	164 (49.8)	177 (36.0)	295 (62.2)	767(87.2)
once a week	107 (66.0)	95 (79.8)	105 (90.5)	60 (37.7)	103 (65.2)	40 (64.5)	43 (67.2)	45 (49.5)	57 (67.1)	208(87.4)
twice a week	37 (56.1)	33 (64.7)	42 (80.8)	22 (33.3)	34 (51.5)	13 (52.0)	17 (68.0)	14 (37.8)	23 (62.2)	77(80.2)
three times a week	30 (56.6)	27 (73.0)	30 (81.1)	18 (35.3)	30 (53.6)	12 (50.0)	11 (47.8)	10 (41.7)	14 (56.0)	73(83.0)
χ^2^	2.81	11.57	3.89	2.20	4.82	14.26	8.98	6.03	1.22	4.97
*p*	.422	.009	.274	.532	.186	.003	.030	.110	.749	.174
**history of MSD**										
No	686 (61.0)	655 (81.0)	699 (86.1)	354 (32.0)	664 (59.7)	166 (43.7)	205 (52.4)	215 (37.9)	340 (62.2)	1304(85.5)
yes	111 (72.5)	102 (80.3)	105 (84.0)	60 (39.7)	100 (66.2)	26 (49.1)	30 (60.0)	31 (40.8)	49 (66.2)	214(95.1)
χ^2^	7.68	0.03	0.39	3.62	2.40	0.54	1.02	0.24	0.46	15.72
*p*	.006	.863	.534	.057	.121	.461	.312	.621	.498	< .001
**BMI**										
< 18.5	126(13.5)	112(12.7)	127(13.5)	62(12.7)	115(12.6)	24(10.9)	30(10.6)	33(10.6)	53(11.2)	183(13.4)
18.5≦BMI<24	592(63.2)	561(63.7)	593(63.0)	297(60.7)	563(61.7)	127(57.5)	169(59.5)	183(59.0)	285(60.0)	835(61.4)
24≦BMI<27	134(14.3)	116(13.2)	131(13.9)	75(15.3)	141(15.5)	42(19.0)	55(19.4)	60(19.4)	79(16.6)	201(14.8)
27≦BMI<30	55(5.9)	58(6.6)	59(6.3)	35(7.2)	62(6.8)	20(9.0)	21(7.4)	21(6.8)	31(6.5)	91(6.7)
BMI≧30	29(3.1	34(3.9)	32(3.4)	20(4.1)	31(3.4)	8(3.6)	9(3.2)	13(4.2)	27(5.7)	51(3.7)
χ^2^	3.5	7.00	1.63	4.50	9.84	11.19	12.15	13.40	19.33	6.51
*p*	.476	.136	.802	.341	.043	.024	.016	.009	.001	.166
**BW (kg)**										
< 45	104(9.6)	101(9.8)	107(9.8)	51(8.9)	94(8.9)	20(7.4)	28(8.2)	29(8.1)	48(8.8)	148(9.5)
45≦BW<55	437(40.2)	404(39.2)	429(39.1)	213(37.2)	413(39.3)	96(35.7)	122(35.8)	124(34.7)	195(35.9)	614(39.5)
55≦BW<65	308(28.3)	282(27.4)	313(28.6)	161(28.1)	297(28.2)	78(29.0)	106(31.1)	117(32.8)	168(30.9)	425(27.3)
65≦BW<75	73(6.7)	72(7.0)	73(6.7)	47(8.2)	86(8.2)	23(8.6)	25(7.3)	30(8.4)	46(8.5)	115(7.4)
BW≧75	166(15.3%)	171(16.6)	174(15.9)	100(17.5)	162(15.4)	52(19.3)	60(17.6)	57(16.0)	86(15.8)	253(16.3)
χ^2^	6.81	2.15.	6.94	6.36	12.13	7.66	7.65	12.80	12.55	11.39
*p*	.146	708	.139	.173	.016	.105	.105	.012	.014	.022

^#^ Each group’s statistics are expressed in terms of number of people those who had musculoskeletal discomfort.

^§^Multiple (more than one) sites musculoskeletal discomfort.

### Association between participants’ demographics, and upper limb muscle discomfort

Upper limb muscle discomfort referred to discomfort of the left elbow joint, right elbow joint, left wrist, or right wrist. A chi-square test was performed to determine the association between participants’ demographics, and upper limb muscle discomfort. The results revealed the following: (1) number of hours worked per day, exercise habits, and BMI were significantly associated with those who experienced left elbow joint discomfort (χ^2^ = 9.47, *p* = .024, χ^2^ = 14.26, *p* = .003, χ^2^ = 11.19, *p* = .024); (2) age, work seniority, seniority in the current unit, exercise habits, and BMI were significantly associated with those who experienced right elbow joint discomfort (χ^2^ = 11.73, *p* = .008, χ^2^ = 12.01, *p* = .007, χ^2^ = 12.53, *p* = .014, χ^2^ = 8.98, *p* = .030, χ^2^ = 12.15, *p* = .016); (3) age, hours standing per day, BMI, and BW were significantly associated with those who experienced left wrist discomfort(χ^2^ = 8.12, *p* = .044, χ^2^ = 12.0, *p* = .017, χ^2^ = 13.40, *p* = .009, χ^2^ = 12.80, *p* = .012); (4) age, work seniority, seniority in the current unit, department type, work mode, BMI, and BW were significantly associated with those who experienced right wrist discomfort (χ^2^ = 23.29, 27.10, 23.33, *p* < .001, χ^2^ = 14.89, *p* = .021, χ^2^ = 8.42, *p* = .038, χ^2^ = 13.40, *p* = .009, χ^2^ = 12.80, *p* = .012); (5) age, work seniority, seniority in the current unit, number of hours worked per day, number of days worked per week, history of MSD, and BW were significantly associated with those who experienced multiple sites discomfort (χ^2^ = 11.74, *p* = .008, χ^2^ = 10.81, *p* = .013, χ^2^ = 25.01, *p* < .001, χ^2^ = 9.56, *p* = .023, χ^2^ = 10.78, *p* = .013, χ^2^ = 15.72, *p* < .001, χ^2^ = 11.39, *p* = .022). (Table 2).

### Association between participants’ demographics, and lower limb muscle discomfort

Lower limb muscle discomfort referred to discomfort of the left knee joint, right knee joint, left ankle joint, right ankle joint, left hip joint, or right hip joint. A chi-square test was performed to determine the association between participants’ demographics, and lower limb muscle discomfort. The results presented in Table 3 indicated the following: (1) age, work seniority, job title, department type, BMI and BW were significantly associated with those who experienced left knee joint discomfort (χ^2^ = 13.62, *p* = .003, χ^2^ = 12.46, *p* = .006, χ^2^ = 9.12, *p* = .028, χ^2^ = 26.43, *p* < .001, χ^2^ = 36.21, *p* < .001, χ^2^ = 25.37, *p* < .001); (2) age, work seniority, seniority in the current unit, department type, BMI and BW were significantly associated with those who experienced right knee joint discomfort (χ^2^ = 10.48, *p* = .015, χ^2^ = 13.06, *p* = .005, χ^2^ = 11.22, *p* = .024, χ^2^ = 18.00, *p* = .006, χ^2^ = 30.04, *p* < .001, χ^2^ = 17.16, *p* = .002); (3) job title, department type, BMI and BW were significantly associated with those who experienced left ankle joint discomfort (χ^2^ = 10.40, *p* = .015, χ^2^ = 13.25, *p* = .039, χ^2^ = 22.42, *p* < .001, χ^2^ = 31.39, *p* < .001); (4) work mode, hours standing per day, BMI and BW were significantly associated with those who experienced right ankle joint discomfort (χ^2^ = 8.44, *p* = .038, χ^2^ = 9.52, *p* = .049, χ^2^ = 31.47, *p* < .001, χ^2^ = 30.33, *p* < .001); (5) age and number of hours worked per day were significantly associated with those who experienced left hip joint discomfort (χ^2^ = 8.46, *p* = .037, χ^2^ = 13.81, *p* = .003); (6) department type was significantly associated with those who experienced right hip joint discomfort (χ^2^ = 15.56, *p* = .016); (7) age, work seniority, seniority in the current unit, job title, department type, work mode, number of hours worked per day, number of days worked per week, exercise habits, history of MSD, BW and BMI were significantly associated with those who multiple sites discomfort (χ^2^ = 97.6, 58.79, 51.34, 55.55, 50.99, 25.72, *p* < .001, χ^2^ = 13.56, *p* = .004, χ^2^ = 23.44, *p* < .001, χ^2^ = 9.74, *p* = .021, χ^2^ = 4.98, *p* = .016, χ^2^ = 34.10, 32.89, *p* < .001).

**Table 3:** Lower limb muscle discomfort in nurses across different the demographic characteristics ^#^.

variables	left knee joint	right knee joint	left ankle joint	right ankle joint	left hip joint	right hip joint	^§^multiple sites
	n = 289 60.1%	n = 292 59.7%	n = 228 47.0%	n = 212 47.3%	n = 195 40.8%	n = 195 41.7%	n = 790 43.8%
**age (years)**							
< 30	11 (42.3)	10 (38.5)	19 (44.2)	15 (44.1)	6 (19.4)	7 (24.1)	212(34.4)
30 to< 40	71 (50.4)	77 (53.8)	80 (49.7)	79 (54.1)	80 (45.7)	78 (46.7)	196(37.3)
40 to<50	82 (64.6)	77 (60.6)	52 (44.1)	44 (39.3)	47 (37.3)	46 (36.5)	218(54.2)
≧50	125 (66.8)	128 (66.3)	77 (47.2)	74 (47.4)	62 (42.5)	64 (43.8)	133(68.6)
χ^2^	13.62	10.48	1.02	5.74	8.46	7.07	97.06
*p*	.003	.015	.798	.125	.037	.070	< .001
**work seniority(years)**							
< 10	42 (47.2)	45 (49.5)	55 (48.2)	54 (52.9)	36 (33.3)	37 (36.3)	336(36.2)
10–19	94 (56.6)	89 (54.3)	88 (48.4)	79 (47.9)	76 (42.0)	74 (42.3)	197(46.4)
20–29	96 (67.6)	92 (64.8)	61 (50.8)	50 (43.1)	46 (38.7)	49 (40.5)	163(55.4)
≧30	57 (67.9)	66 (71.7)	24 (34.8)	29 (44.6)	37 (52.9)	35 (50.0)	61(67.0)
χ^2^	12.46	13.06	5.05	2.33	7.04	3.32	58.79
*p*	.006	.005	.168	.506	.071	.345	< .001
**seniority in the current unit (years)**							
<1	19 (46.3)	20 (50.0)	24 (46.2)	22 (50.0)	16 (30.8)	16 (33.3)	73(31.3)
1 to< 3	35 (56.5)	36 (55.4)	32 (47.1)	36 (58.1)	29 (41.4)	29 (42.6)	96(34.3)
3 to< 5	37 (55.2)	34 (52.3)	34 (46.6)	31 (44.3)	26 (36.1)	29 (40.3)	111(43.5)
5 to< 10	44 (55.7)	42 (51.9)	43 (47.8)	33 (41.8)	41 (45.6)	37 (41.6)	136(41.0)
≧10	154 (66.4)	160 (67.2)	95 (47.0)	90 (46.6)	83 (42.8)	84 (44.0)	374(53.2)
χ^2^	8.70	11.22	0.04	4.27	3.99	1.88	51.34
*p*	.069	.024	1.000	.371	.407	.759	< .001
**job title**							
administrative supervisor	10 (37.0)	15 (53.6)	5 (19.2)	6 (26.1)	12 (44.4)	12 (44.4)	71(50.7)
full-time nurses	195 (60.0)	195 (58.9)	158 (46.7)	148 (48.1)	148 (42.8)	145 (43.2)	487(39.4)
part-time nurses	15 (78.9)	14 (73.7)	11 (50.0)	10 (50.0)	6 (23.1)	7 (29.2)	60(40.8)
Other	69 (62.7)	68 (61.3)	54 (54.5)	48 (49.5)	29 (36.7)	31 (38.3)	141(65.9)
χ^2^	9.12	2.18	10.40	4.47	4.64	2.32	55.55
*p*	.028	.536	.015	.215	.200	.509	< .001
**department type**							
internal medicine	74 (60.7)	73 (60.3)	65 (50.4)	49 (45.8)	58 (43.0)	51 (39.5)	190(43.0)
surgical department	73 (68.2)	71 (66.4)	65 (57.0)	58 (50.9)	56 (48.3)	62 (56.4)	112(45.3)
obstetrics & Gynecology	15 (41.7)	18 (50.0)	12 (37.5)	13 (46.4)	11 (34.4)	12 (38.7)	37(34.3)
intensive care unit	28 (43.8)	31 (45.6)	19 (33.3)	28 (48.3)	21 (33.3)	19 (30.6)	76(35.8)
pediatric department	32 (55.2)	29 (49.2)	28 (47.5)	27 (49.1)	22 (39.3)	22 (38.6)	96(33.4)
other more than 2 department	65 (74.7)	66 (72.5)	37 (43.5)	35 (44.3)	26 (39.4)	27 (39.1)	259(56.2)
χ^2^	26.43	18.00	13.25	2.05	8.98	15.56	50.99
*p*	< .001	.006	.039	.915	.175	.016	< .001
**work mode**							
Fixed	97 (59.9)	103 (60.6)	57 (39.0)	52 (37.4)	51 (35.2)	53 (37.1)	294(51.9)
three-shift rotation (8 hours/day)	170 (59.6)	168 (58.5)	151 (49.8)	139 (51.1)	130 (43.0)	130 (43.9)	425(39.1)
Other	20 (66.7)	19 (67.9)	19 (59.4)	19 (57.6)	14 (46.7)	12 (42.9)	11(42.3)
More than two work mode	2 (50.0)	2 (50.0)	1 (25.0)	2 (50.0)	0 (0.0)	0 (0.0)	32(50)
χ^2^	0.74	1.15	7.43	8.44	3.65	2.60	25.72
*p*	.864	.765	.059	.038	.302	.458	< .001
**number of hours worked per day (hour)**							
≦7	3 (50.0)	2 (33.3)	0 (0.0)	1 (25.0)	2 (66.7)	2 (66.7)	44(44.4)
8	122 (58.7)	122 (58.7)	86 (43.4)	81 (42.9)	54 (31.0)	62 (36.3)	327(47.3)
9 to 11	141 (61.0)	141 (59.0)	120 (49.8)	106 (49.3)	118 (44.5)	111 (42.9)	338(39.5)
≧12	23 (63.9)	27 (75.0)	22 (52.4)	24 (60.0)	21 (58.3)	20 (57.1)	81(51.6)
χ^2^	.74	5.38	5.80	5.23	13.81	6.43	13.56
*p*	.864	.146	.122	.156	.003	.092	.004
**standing (hour/ day)**							
1–4	75 (62.5)	72 (57.1)	40 (37.7)	36 (37.1)	35 (34.3)	37 (37.4)	199(44.8)
5	28 (54.9)	30 (57.7)	22 (44.0)	21 (44.7)	25 (41.7)	25 (43.1)	86(38.7)
6–7	126 (59.4)	128 (60.7)	109 (49.8)	103 (50.5)	87 (41.8)	82 (40.4)	513(45.4)
8	38 (61.3)	38 (60.3)	30 (44.8)	26 (44.1)	23 (34.8)	30 (43.5)	101(43.7)
≧9	22 (61.1)	24 (64.9)	27 (62.8)	26 (63.4)	25 (59.5)	21 (53.8)	89(42.0)
χ^2^	0.95	0.93	8.95	9.52	8.95	3.41	3.49
*p*	.917	.920	.062	.049	.062	.492	.479
**number of days worked per week (day)**							
1–3	13 (56.5)	11 (47.8)	10 (50.0)	10 (47.6)	10 (43.5)	10 (45.5)	38(34.2)
4	10 (55.6)	10 (58.8)	12 (52.2)	11 (57.9)	7 (28.0)	9 (37.5)	48(36.4)
5	153 (59.3)	156 (59.3)	113 (43.3)	114 (47.5)	98 (38.4)	100 (40.0)	364(41.1)
6–7	113 (62.1)	115 (61.8)	93 (51.4)	77 (45.8)	80 (45.7)	76 (44.2)	307(51.3)
χ^2^	0.65	1.72	3.15	1.01	4.11	1.04	23.44
*p*	.886	.633	.369	.800	.250	.792	< .001
**exercise habits**							
rarely	209 (58.9)	207 (57.7)	168 (46.4)	153 (45.7)	155 (41.8)	150 (41.4)	560(41.9)
once a week	46 (63.9)	45 (63.4)	33 (46.5)	34 (54.8)	24 (38.1)	26 (43.3)	115(48.3)
twice a week	19 (70.4)	21 (72.4)	15 (57.7)	12 (46.2)	10 (37.0)	11 (40.7)	46(47.9)
three times a week	15 (55.6)	19 (63.3)	12 (46.2)	13 (52.0)	6 (35.3)	8 (42.1)	49(55.7)
χ^2^	2.07	3.13	1.26	2.00	0.71	0.09	9.74
*p*	.557	.371	.739	.571	.871	.993	.021
**history of MSD**							
No	257 (60.0)	254 (58.5)	191 (45.9)	179 (46.7)	171 (40.4)	173 (42.1)	652(42.8)
Yes	32 (60.4)	38 (69.1)	37 (53.6)	33 (50.8)	24 (43.6)	22 (38.6)	114(50.7)
χ^2^	0.00	2.27	1.41	0.36	0.21	0.25	4.98
*p*	.963	.132	.235	.547	.649	.616	.016
**BMI**							
< 18.5	30(8.3)	27(7.5)	23(8.7)	22(8.6)	28(12.0)	30(12.9)	66(9.8)
18.5≦BMI<24	210(58.3)	220(61.5)	151(57.4)	142(55.3)	143(61.4)	143(61.4)	405(59.9)
24≦BMI<27	79(21.9)	73(20.4)	51(19.4)	52(20.2)	40(17.2)	38(16.3)	124(18.3)
27≦BMI<30	26(7.2)	26(7.3)	24(9.1)	28(10.9)	15(6.4)	15(6.4)	53(7.8)
BMI≧30	15(4.2)	12(3.4)	14(5.3)	13(5.1)	7(3.0)	7(3.0)	28(4.1)
χ^2^	36.21	30.04	22.42	31.47	3.34	1.75	34.10
*p*	< .001	< .001	< .001	< .001	.502	.781	< .001
**BW (kg)**							
< 45	31(7.2)	34(8.1)	16(5.1)	22(7.4)	28(10.0)	30(10.9)	59(7.5)
45≦BW<55	141(32.9)	143(33.9)	99(31.6)	89(29.9)	103(36.7)	101(36.6)	284(35.9)
55≦BW<65	133(31.0)	135(32.0)	105(33.5)	101(33.9)	81(28.8)	83(30.1)	230(29.1)
65≦BW<75	42(9.8)	38(9.0)	32(10.2)	35(11.7)	17(6.0)	17(6.2)	73(9.2)
BW≧75	82(19.1)	72(17.1)	61(19.5)	51(17.1)	52(18.5)	45(16.3)	144(18.2)
χ^2^	25.37	17.16	31.39	30.33	3.47	3.15	32.89
*p*	< .001	.002	< .001	< .001	.482	.532	< .001

^#^ Each group’s statistics are expressed in terms of number of people those who had musculoskeletal discomfort.

^§^Multiple (more than one) sites musculoskeletal discomfort.

### Risk factors for shoulder and neck/back MSD in nurses

In this study, we used a logistic regression to identify the potential risk factors of discomfort in the shoulder and neck/back, which was more common than discomfort in other locations among the research participants.

#### Risk factors for shoulder discomfort

In this study, shoulder discomfort referred to discomfort of the left or right shoulder. The results presented in Table 4 indicated that risk factors for left shoulder discomfort included “department type”and “exercise habits“. Further comparison suggested that regarding “department type“, nurses in the “surgical department” (OR = 1.93; *p* = .023) had a higher risk than those in the “internal medicine” department and that those in the “intensive care unit” department (OR = 0.52; *p* = .015) had a lower risk than those in the “internal medicine” department. Regarding “exercise habits“, participants who “exercised twice a week” (OR = 0.46; *p* = .019) had a lower risk of experiencing shoulder discomfort than those who “rarely” exercised habits. Risk factors for right shoulder discomfort included “age“. Further comparison indicated that participants who were ages “30 to < 40 years” (OR = 2.42; *p* = .005), “40 to <50 years” (OR = 2.34; *p* = .010), or “≧50 years” (OR = 2.10; *p* = .023) had a higher risk of experiencing right shoulder discomfort than those less than 30 years old.

**Table 4:** Risk factor for shoulder discomfort in nurses.

Items	B	Odds ratio	95% CI of OR	*p*
**Left shoulder**				
Constant	1.88	6.56		.122
**age**^§^				
30 to< 40	0.33	1.40	0.69–2.84	.357
40 to<50	0.21	1.24	0.51–3.02	.640
≧50 vs	0.36	1.43	0.49–4.16	.510
**work seniority** ^†^				
10–19	0.31	1.36	0.81–2.30	.247
20–29	0.43	1.54	0.75–3.16	.240
≧30	-0.56	0.57	0.24–1.38	.214
**job title** ^¶^				
full-time nurses	-0.01	0.99	0.45–2.21	.987
part-time nurses	-0.17	0.85	0.29–2.45	.757
Other	-0.66	0.52	0.20–1.36	.181
**department type** ^#^				
surgical department	0.66	1.93	1.10–3.41	.023
obstetrics & Gynecology	-0.01	0.99	0.49–2.01	.979
intensive care unit	-0.65	0.52	0.31–0.88	.015
pediatric department	0.08	1.08	0.60–1.97	.789
Other	0.12	1.13	0.63–2.01	.681
more than 2 department	-0.78	0.46	0.18–1.18	.107
**number of hours worked per day** ^⌖^				
8	-0.88	0.41	0.05–3.53	.421
9 to 11	-0.83	0.44	0.05–3.70	.447
≧12	-0.39	0.68	0.07–6.58	.739
**exercise habits** ^□^				
once a week	0.03	1.03	0.62–1.73	.903
twice a week	-0.78	0.46	0.24–0.88	.019
three times a week	-0.28	0.75	0.33–1.69	.493
**Right shoulder**				
Constant	0.54	1.71		.525
**age**^§^				
30 to< 40	0.88	2.42	1.31–4.47	.005
40 to<50	0.85	2.34	1.22–4.48	.010
≧50	0.74	2.10	1.11–3.99	.023
**number of hours worked per day** ^⌖^				
8	0.21	1.23	0.25–6.19	.798
9 to 11	0.69	1.99	0.40–9.93	.402
≧12	1.15	3.15	0.52–19.00	.210

^§^ Reference: < 30;

^†^ Reference: < 10;

^¶^ Reference: administrative supervisor;

^#^ Reference: internal medicine;

^⌖^ Reference: ≦7;

^□^ Reference: rarely;

^⌘^ Reference: <1;

^✞^ Reference:1–4;

^✡^ Reference: fixed;

^⟡^ Reference:1–3.

#### Risk factors for neck/back discomfort

The logistic regression results presented in Table 5 revealed that risk factors for neck discomfort included “seniority in the current unit,” “job title,” and “history of MSD”. Further comparison indicated that regarding “seniority in the current unit,” participants with experience of “1 to < 3 years” (OR = 1.68; *p* = .025) or “≧10 years” (OR = 1.89; *p* = .051) had a higher risk of experiencing neck discomfort than those with experience of “<1 year” in their current unit. Regarding “job title,” “full-time nurses” (OR = 0.46; *p* = .044), “part-time nurses” (OR = 0.32; *p* = .010), and “other” (OR = 0.34; *p* = .044) had a lower risk of experiencing neck discomfort than “nursing supervisors.” Regarding “history of MDS” participants with a history of injury (OR = 1.95; *p* < .001) had a higher risk of experiencing neck discomfort than those who had no history of MDS.

**Table 5:** Risk factor for neck and back discomfort in nurses.

Item	B	Odds ratio	95% CI of OR	*p*
**Neck discomfort**				
Constant	0.04	1.04		.971
**age**^§^				
30 to< 40	0.29	1.34	0.81–2.22	.255
40 to<50	0.17	1.18	0.63–2.22	.607
≧50	0.56	1.76	0.80–3.86	.159
**work seniority** ^†^				
10–19	0.40	1.49	0.82–2.70	.186
20–29	0.42	1.52	0.78–2.93	.217
≧30	-0.30	0.74	0.33–1.65	.467
**seniority in the current unit** ^⌘^				
1 to< 3	0.52	1.68	1.07–2.66	.025
3 to< 5	0.18	1.20	0.61–2.36	.597
5 to< 10	0.33	1.39	0.72–2.71	.330
≧10	0.64	1.89	1.00–3.56	.050
**job title** ^¶^				
full-time nurses	-0.78	0.46	0.21–0.98	.044
part-time nurses	-1.15	0.32	0.13–0.76	.010
Other	-1.07	0.34	0.12–0.97	.044
**number of hours worked per day** ^⌖^				
8	-0.84	0.43	0.11–1.74	.238
9 to 11	-0.27	0.76	0.19–3.08	.705
≧12	-0.26	0.77	0.18–3.40	.734
**Standing (hour/ day)**^✞^				
5	0.28	1.32	0.86–2.03	.207
6–7	0.12	1.13	0.81–1.57	.471
8	-0.30	0.74	0.48–1.14	.173
≧9	0.26	1.30	0.75–2.24	.353
**history of MSD** (yes vs no)	0.67	1.95	1.31–2.91	< .001
**Upper back discomfort**				
Constant	-1.73	0.18		< .001
**age**^§^				
30 to< 40	0.51	1.66	0.92–3.01	.094
40 to<50	0.52	1.68	0.84–3.33	.140
≧50	0.81	2.24	1.07–4.68	.033
**work seniority** ^†^				
10–19	0.18	1.19	0.66–2.16	.563
20–29	-0.15	0.86	0.45–1.64	.642
≧30	-0.47	0.63	0.29–1.34	.228
**seniority in the current unit** ^⌘^				
1 to< 3	0.34	1.41	0.84–2.36	.191
3 to< 5	0.31	1.37	0.68–2.75	.383
5 to< 10	0.39	1.48	0.74–2.94	.264
≧10	0.79	2.19	1.15–4.18	.017
**department type** ^#^				
surgical department	0.19	1.21	0.84–1.73	.313
obstetrics & Gynecology	0.39	1.48	0.90–2.44	.124
intensive care unit	-0.27	0.77	0.51–1.16	.207
pediatric department	0.07	1.08	0.72–1.62	.722
Other	0.06	1.06	0.70–1.60	.778
more than 2 department	-0.60	0.55	0.23–1.32	.182
**work mode** ^✡^				
three-shift rotation (8 hours/day)	-0.07	0.93	0.68–1.28	.654
Other	0.54	1.71	0.95–3.07	.072
more than two work mode	0.80	2.23	0.34–14.62	.402
**Lower back discomfort**				
Constant	-0.10	0.90		.890
**age**^§^				
30 to< 40	0.23	1.26	0.78–2.06	.348
40 to<50	0.04	1.04	0.57–1.89	.897
≧50	0.23	1.26	0.64–2.45	.504
**work seniority** ^†^				
10–19	0.31	1.36	0.77–2.39	.291
20–29	0.54	1.72	0.92–3.19	.089
≧30	-0.27	0.76	0.37–1.57	.460
**seniority in the current unit** ^⌘^				
1 to< 3	0.52	1.68	1.07–2.62	.024
3 to< 5	0.18	1.19	0.62–2.28	.594
5 to< 10	0.09	1.10	0.58–2.06	.777
≧10	0.46	1.58	0.88–2.83	.127
**department type** ^#^				
surgical department	-0.06	0.94	0.66–1.35	.744
obstetrics & Gynecology	-0.47	0.63	0.38–1.03	.065
intensive care unit	-0.40	0.67	0.46–0.98	.040
pediatric department	-0.47	0.62	0.42–0.93	.021
Other	0.08	1.08	0.71–1.66	.716
above 2 department	-0.54	0.58	0.27–1.23	.156
**number of hours worked per day** ^⌖^				
8	0.14	1.15	0.32–4.12	.826
9 to 11	0.55	1.73	0.48–6.16	.399
≧12	0.92	2.51	0.65–9.65	.180
**number of days worked per week**^⟡^				
4	-0.98	0.38	0.19–0.75	.006
5	-0.56	0.57	0.33–0.98	.042
6–7	-0.23	0.79	0.45–1.39	.422

^†^ Reference: < 10;

^¶^ Reference: administrative supervisor;

^#^ Reference: internal medicine;

^⌖^ Reference: ≦7;

^□^ Reference: rarely;

^⌘^ Reference: <1;

^✞^ Reference:1–4;

^✡^ Reference: fixed;

^⟡^ Reference:1–3.

Logistic regression results indicated that risk factors for upper back discomfort included “age” and “seniority in the current unit”. Further comparison revealed that in terms of “age,” participants who were “≧50 years” (OR = 2.24; *p* = .033) had a higher risk of upper back discomfort than those who were “< 30 years”. Regarding “seniority in the current unit,” participants with “≧10” (OR = 2.19; *p* = .017) of work experience had a higher risk than those with “< 1 year” of experience (Table 5).

Logistic regression results revealed that risk factors for lower back discomfort included “seniority in the current unit,” “department type,” and “number of days worked per week”. Further comparison indicated that regarding “seniority in the current unit,” participants with “1 to 3 years” (OR = 1.68; *p* = .024) of work experience had a higher risk of lower back discomfort than those with “< 1 year” of work experience in their current unit. Regarding “department type,” participants working in the “intensive care unit” department (OR = 0.67; *p* = .040) or “pediatric department” (OR = 0.62; *p* = .021) had a lower risk of lower back discomfort than participants in the “internal medicine” department. Regarding “number of days worked per week,” participants who worked “4 days” (OR = 0.38; *p* = .006) or “5 days” (OR = 0.57; *p* = .042) had a lower risk of lower back discomfort than those who worked “1–3 days.”

## Discussion

In this study, we categorized the MSDs of nurses according to nine locations: the shoulder, neck, lower back, upper back, elbow joint, wrist, knee joint, hip joint, and ankle joint. The shoulder, elbow joint, wrist, knee joint, hip joint, and ankle joint were further divided into the left and right side. Numerous studies have indicated that shoulder, neck, and lower back discomfort are health problems commonly seen in nurses [3, 11, 16, 32,33] The results of the present study indicated that the body locations where discomfort was most commonly felt among participants was, in descending order, the right shoulder, left shoulder, neck, and lower back, with prevalence rates of 85.8%, 80.9%, 62.4%, and 60.4%, respectively. This result is similar to those of many other studies. Lee et al. [10] conducted a study on 386 workers with self-perceived musculoskeletal discomfort. Their results revealed that 303 (78.9%), 268 (70%), and 240 (62.7%) participants had self-perceived shoulder, neck, and lower back or waist discomfort, respectively. Hsieh et al. [17] investigated work environment health and safety situational awareness and discovered that 41.31%, 32.25%, and 31.03% of participants experienced shoulder, neck, and lower back or waist discomfort, respectively. Furthermore, Smith, Mihashi, Adachi, Koga, and Ishitake [34] found that the most common location for discomfort was the shoulder, with 71.9% of Japanese nurses experiencing shoulder discomfort. Yang et al. [35] explored factors related to musculoskeletal discomfort among 341 home care service personnel and found that the shoulder (67.4%) had the highest occurrence rate for musculoskeletal discomfort.

In this study, the neck, shoulder, and wrist were the body locations where the highest number of interviewees felt discomfort. Causal analysis indicated that as the use of computers in hospitals has become more common, nurses have begun spending more time using computers, and ergonomic factors such as computer use posture, suitability of computer tables and chairs, and the mouse have become increasingly important. In addition, there are currently no appropriate personal protective methods for shoulder and neck pain for clinical nurses. Only some work procedures or nursing station work platforms have implemented engineering improvements to correct prolonged improper work posture. Such measures can reduce the prevalence of shoulder and neck pain among clinical nurses. However, some studies have published results that differ from those of this study. Cheng et al. [19] discovered that when nurses had MSDs caused by turning and transferring patients, the locations with the highest prevalence rates were the lower back (77.2%), neck (64.2%), and shoulder (58.7%). This difference in results may be attributable to the fact that Chen et al. [18] focused on MSDs in nurses who moved or transferred patients, whereas the present study focused on nurses in all departments.

Kalkim et al. [16] conducted an investigation of 498 nurses with MSDs and discovered that the body locations with the highest prevalence rates were the lower back (78.5%), back (74.9%), knee joint (63.1%), neck (61.2%), and shoulder (59.6%). Although lower back discomfort was not the most common location of discomfort in the present study, it nevertheless affected 60.4% of the participants. The participants in this study worked 8.9 ± 1.51 hours per day, and 40.38% of them had daily rest time. The participants in the study conducted by Kalkim et al. [16] indicated that worked ≥9 hours per day and did not have daily rest time. This may have caused the difference in results for the different MSD locations. Another result of this study indicated that in the correlation between personal attributes and MSDs, most MSD locations were related to age. Age was a predictor of right shoulder and upper back discomfort, with nurses aged 30 years (including) or older having a higher risk than those who were younger than 30 years. The results of this study differ from those reported by Yang et al. [35], who discovered that younger service personnel were at higher risk. The reason may be because younger service personnel have less refined nursing skills and less precise care actions, resulting in higher risk of waist pain.

This study found that seniority level was significantly correlated with left and right knee joint discomfort and that participants with 30 years of work experience or more were more likely to have knee joint discomfort. Furthermore, seniority in the current unit was a predictor of neck and back discomfort as well as upper and lower back discomfort. This study also discovered that nursing seniority and seniority in the current unit were significantly correlated with neck, upper and lower back, and right elbow joint discomfort. This result reflects what was reported by Tinubu, Mbada, Oyeyemi, and Fabunmi [15], which indicated that nurses with more than 20 years of clinical experience were more likely to have work-related MSD than were those with 11–20 years of experience. Furthermore, the department type that the participants worked in was correlated to most shoulder/neck/back, upper limb, and lower limb MSD locations, with MSD prevalence being highest among participants who worked in the surgical department. Department type was also a predictor of left shoulder discomfort and lower back discomfort. Surgical department workers are more likely to move and transport patients before and after surgery. The results of the present study reflect the results reported by Coggon, Inskip, Croft, Campbell, and Cooper [36], who proposed that when moving heavy objects, mechanical stress is concentrated in the hip joint, knee joint, and fingers. The increased stress on these joints also increases the likelihood of injury. A possible explanation for this situation is that the participants are a lot of dropouts of this study and can be a limitation of the study. However, Kjellberg et al. [37] pointed out that back disorders among nursing personnel are associated with the work task of assisting patients during transfers. Thus, it was evident that poor work techniques affected MSD in this study. Epidemiological studies that have demonstrated that high BMI is linked to MSD have not revealed factors that explain this link [21]. The current study revealed a significant correlation between the BMI and MSDs development among nurses. This finding was consistent with several previous studies [21,22] who stated the positive association between BMI and MSDs. Nevertheless, the finding was contradictory with a study by Tantawy et al. [2], which reporting no significant correlation between MSDs and BMI among Ahlia University students in different disciplines.

## Conclusion

This study investigated MSD among nurses and determined the prevalence of musculoskeletal discomfort for different parts of the body. We used logistic regression to analyze discomfort in the shoulder as well as in the neck and back, which were the body locations with relatively high prevalence rates. Different locations had different predictors for discomfort. We discovered that nurses had a higher incidence of musculoskeletal discomfort and that differences in work practices and conditions corresponded to different locations of discomfort. We recommend that nursing supervisors provide specialized training for different types of nursing. Determining the risk factors in a hospital environment and implementing measure for ergonomic improvement and to ensure appropriate work postures and methods can prevent or reduce the incidence of MSDs in nurses.

The main limitation of the present study is the use of a questionnaire among nurses at a certain medical center. Data regarding the occurrence of musculoskeletal discomfort was based on self-reported information provided by participants, and no physiological testing was conducted to confirm the diagnosis. Thus, musculoskeletal discomfort caused by non-work factors cannot be eliminated, meaning that the prevalence of musculoskeletal discomfort reported in this study may be overestimated. Also, the muscular discomfort is for multiple reasons and not for the workload. In addition, the present study only investigated demographic characteristics and work-related factors associated with musculoskeletal discomfort neglecting other factors that possibly may influence development of the condition, such as burnout, resilience, satisfaction, work stress level, and the level of training that nurses have received on how to perform the mobilizations when transferring patients. We recommend that future studies increase the number of participants recruited and use random sampling or stratified sampling to collect data. Physiological measurements can be implemented to conduct a more comprehensive study and thereby obtain more objective and accurate data verification and results. This can better reflect influential factors of musculoskeletal discomfort among nursing personnel and make the sample more representative.

## Supporting information

S1 File 肌肉骨骼不適症狀 [Musculoskeletal disorder] (*original Chinese version*). (PDF)

S2 File Dataset. (SAV)

S3 File STROBE statement. (DOC)
