# Supplementary material for: Exploring the factors affecting musculoskeletal disorders risk among hospital nurses
Source: PLoS One. 2020 Apr 16;15(4):e0231319. doi: 10.1371/journal.pone.0231319 (PMC7162448; doi:10.1371/journal.pone.0231319)
Supplement: S1 File — (PDF) [file pone.0231319.s001.pdf]

貳、肌肉骨骼不適症狀

第一部份：脖子

右側陰影區標示出脖子所指的範圍，您在最近一年內右側陰影區標示出脖子所指的範圍，有沒有疼痛、酸痛、發麻、刺痛或任何不舒服的感覺出現？

① 沒有 ，本頁不需回答，請跳至下頁。
② 有 ，請您繼續詳填下列問題。

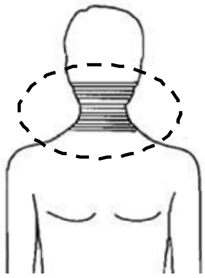

1.您的症狀出現的時間為？

① 現在 ② 過去一個月 ③ 過去半年中 ④ 過去一年中

2.您的症狀持續多久了？

① 1個月 ② 3個月 ③ 6個月 ④ 1年 ⑤ 3年 ⑥ 3年以上

3.您的症狀為(可重複選擇) ？

① 酸痛 ② 紅腫 ③ 發麻 ④ 刺痛 ⑤ 半夜痛醒 ⑥ 肌肉萎縮
⑦ 其他________________________（請說明）

4.您的症狀對您的影響為？

① 完全不影響生活與工作 ② 稍微降低工作能力 ③ 工作能力明顯降低
④ 曾因此請假休養 ⑤ 連生活都受到影響 ⑥ 完全不能動作
⑦ 其他________________________（請說明）

5.您的症狀出現頻率為？

① 幾乎每天出現 ② 約一星期一次 ③ 約一個月一次 ④ 約半年一次
⑤ 半年以上才出現一次

6.您是否曾尋求治療？

① 未予理會（請跳答第8題） ② 自行處理 ③ 中醫 ④ 西醫 ⑤ 中西醫

7.治療方法為(可複選)

① 曾動手術 ② 曾復健 ③ 按摩(推拿) ④ 冷熱敷 ⑤ 吃藥 ⑥ 敷藥
⑦ 其他________________________（請說明）

8.您認為造成這些症狀的原因與目前的工作有關嗎？

① 全因工作造成的 ② 一部份與工作有關
③ 不清楚 ④ 與工作無關，原因是____________________（請說明）

9. 請**塗黑**您脖子範圍的疼痛、酸痛、發麻、刺痛或任何不舒服的感覺出現嚴重程度。

一點也不 非常嚴重

① ② ③ ④ ⑤ ⑥ ⑦ ⑧ ⑨ ⑩

#-7-#

第二部份：肩膀

右側陰影區標示出肩膀所指的範圍，您在最近一年內右側陰影區標示出肩膀所指的範圍，有沒有疼痛、酸痛、發麻、刺痛或任何不舒服的感覺出現？

① 都沒有 ，本頁不需回答，請跳至下頁。

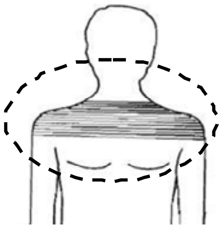

左 肩 ① 沒有 ，本欄不需填寫。
② 有 ，請您務必詳填下列問題。

1.您的症狀出現的時間為？

① 現在 ② 過去一個月
③ 過去半年中 ④ 過去一年中

2.您的症狀持續多久了？

① 1個月 ② 3個月 ③ 6個月
④ 1年 ⑤ 3年 ⑥ 3年以上

3.您的症狀為(可重複選擇)？

① 酸痛 ② 紅腫 ③ 發麻
④ 刺痛 ⑤ 半夜痛醒 ⑥ 肌肉萎縮
⑦ 其他__________________（請說明）

4.您的症狀對您的影響為？

① 完全不影響生活與工作
② 稍微降低工作能力
③ 工作能力明顯降低
④ 曾因此請假休養
⑤ 連生活都受到影響
⑥ 完全不能動作
⑦ 其他__________________（請說明）

5.您的症狀出現頻率為？

① 幾乎每天出現 ② 約一星期一次
③ 約一個月一次 ④ 約半年一次
⑤ 半年以上才出現一次

6.您是否曾尋求治療？

① 未予理會（請跳答第8題） ② 自行處理

③ 中醫 ④ 西醫 ⑤ 中西醫

7.治療方法為(可重複選擇)

① 曾動手術 ② 復健 ③ 按摩(推拿)
④ 冷熱敷 ⑤ 吃藥 ⑥ 敷藥
⑦ 其他 __________________（請說明）

右 肩 ① 沒有 ，本欄不需填寫。
② 有 ，請您務必詳填下列問題。

1.您的症狀出現的時間為？

① 現在 ② 過去一個月
③ 過去半年中 ④ 過去一年中

2.您的症狀持續多久了？

① 1個月 ② 3個月 ③ 6個月
④ 1年 ⑤ 3年 ⑥ 3年以上

3.您的症狀為(可重複選擇)？

① 酸痛 ② 紅腫 ③ 發麻
④ 刺痛 ⑤ 半夜痛醒 ⑥ 肌肉萎縮
⑦ 其他__________________（請說明）

4.您的症狀對您的影響為？

① 完全不影響生活與工作
② 稍微降低工作能力
③ 工作能力明顯降低
④ 曾因此請假休養
⑤ 連生活都受到影響
⑥ 完全不能動作
⑦ 其他__________________（請說明）

5.您的症狀出現頻率為？

① 幾乎每天出現 ② 約一星期一次
③ 約一個月一次 ④ 約半年一次
⑤ 半年以上才出現一次

6.您是否曾尋求治療？

① 未予理會（請跳答第8題） ② 自行處理

③ 中醫 ④ 西醫 ⑤ 中西醫

7.治療方法為(可重複選擇)

① 曾動手術 ② 曾復健 ③ 按摩(推拿)
④ 冷熱敷 ⑤ 吃藥 ⑥ 敷藥
⑦ 其他 __________________（請說明）

8.您認為造成這些症狀的原因與目前的工作有關嗎？

① 全因工作造成的
② 一部份與工作有關
③ 不清楚
④ 與工作無關，原因是（請說明）

____

9. 請**塗黑**您左肩膀範圍的疼痛、酸痛、發麻、刺痛或任何不舒服的感覺出現嚴重程度。

一點也不　　　　　　　　　　　　非常嚴重

① ② ③ ④ ⑤ ⑥ ⑦ ⑧ ⑨ ⑩

8.您認為造成這些症狀的原因與目前的工作有關嗎？

① 全因工作造成的
② 一部份與工作有關
③ 不清楚
④ 與工作無關，原因是（請說明）

____

9. 請**塗黑**您右肩膀範圍的疼痛、酸痛、發麻、刺痛或任何不舒服的感覺出現嚴重程度。

一點也不　　　　　　　　　　　　非常嚴重

① ② ③ ④ ⑤ ⑥ ⑦ ⑧ ⑨ ⑩

第三部份：上背

右側陰影區標示出上背所指的範圍，您在最近一年內右側陰影區標示出上背所指的範圍，有沒有疼痛、酸痛、發麻、刺痛或任何不舒服的感覺出現？

① 沒有　，本頁不需回答，請跳至下頁。
② 有　　，請您繼續詳填下列問題。

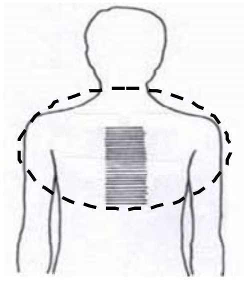

1.您的症狀出現的時間為？

① 現在　② 過去一個月　③ 過去半年中　④ 過去一年中

2.您的症狀持續多久了？

① 1個月　② 3個月　③ 6個月　④ 1年　⑤ 3年　⑥ 3年以上

3.您的症狀為(可重複選擇)？

① 酸痛　② 紅腫　③ 發麻　④ 刺痛　⑤ 半夜痛醒　⑥ 肌肉萎縮
⑦ 其他______________________________（請說明）

4.您的症狀對您的影響為？

① 完全不影響生活與工作　② 稍微降低工作能力　③ 工作能力明顯降低
④ 曾因此請假休養　⑤ 連生活都受到影響　⑥ 完全不能動作
⑦ 其他______________________________（請說明）

5.您的症狀出現頻率為？

① 幾乎每天出現　② 約一星期一次　③ 約一個月一次　④ 約半年一次
⑤ 半年以上才出現一次

#-9-#

6.您是否曾尋求治療？

① 未予理會（請跳答第8題） ② 自行處理 ③ 中醫 ④ 西醫 ⑤ 中西醫

7.治療方法為(可複選)

① 曾動手術 ② 曾復健 ③ 按摩(推拿) ④ 冷熱敷 ⑤ 吃藥 ⑥ 敷藥
⑦ 其他____（請說明）

8.您認為造成這些症狀的原因與目前的工作有關嗎？

① 全因工作造成的
② 一部份與工作有關
③ 不清楚
④ 與工作無關，原因是______________________________（請說明）

9.請**塗黑**您上背範圍的疼痛、酸痛、發麻、刺痛或任何不舒服的感覺出現嚴重程度。

一點也不 —— 非常嚴重

① ② ③ ④ ⑤ ⑥ ⑦ ⑧ ⑨ ⑩

第四部份：下背

右側陰影區標示出下背所指的範圍，您在最近一年內右側陰影區標示出下背所指的範圍，有沒有疼痛、酸痛、發麻、刺痛或任何不舒服的感覺出現？

① 沒有 ，本頁不需回答，請跳至下頁。
② 有 ，請您繼續詳填下列問題。

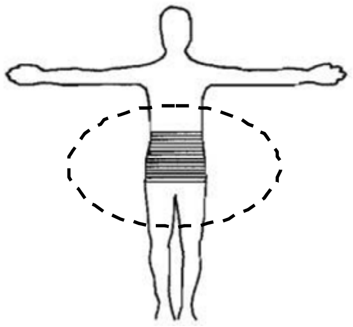

1.您的症狀出現的時間為？

① 現在 ② 過去一個月 ③ 過去半年中 ④ 過去一年中

2.您的症狀持續多久了？

① 1個月 ② 3個月 ③ 6個月 ④ 1年 ⑤ 3年 ⑥ 3年以上

3.您的症狀為(可重複選擇) ？

① 酸痛 ② 紅腫 ③ 發麻 ④ 刺痛 ⑤ 半夜痛醒 ⑥ 肌肉萎縮
⑦ 其他____（請說明）

4.您的症狀對您的影響為？

① 完全不影響生活與工作 ② 稍微降低工作能力 ③ 工作能力明顯降低
④ 曾因此請假休養 ⑤ 連生活都受到影響 ⑥ 完全不能動作
⑦ 其他____（請說明）

#-:-#

5.您的症狀出現頻率為？

① 幾乎每天出現 ② 約一星期一次 ③ 約一個月一次 ④ 約半年一次
⑤ 半年以上才出現一次

6.您是否曾尋求治療？

① 未予理會（請跳答第8題） ② 自行處理 ③ 中醫 ④ 西醫 ⑤ 中西醫

7.治療方法為(可複選)

① 曾動手術 ② 曾復健 ③ 按摩(推拿) ④ 冷熱敷 ⑤ 吃藥 ⑥ 敷藥
⑦ 其他＿＿＿＿＿＿＿＿＿＿＿＿＿＿＿＿＿＿＿＿＿＿（請說明）

8.您認為造成這些症狀的原因與目前的工作有關嗎？

① 全因工作造成的
② 一部份與工作有關
③ 不清楚
④ 與工作無關，原因是＿＿＿＿＿＿＿＿＿＿＿＿＿＿＿＿＿（請說明）

9.請**塗黑**您下背範圍的疼痛、酸痛、發麻、刺痛或任何不舒服的感覺出現嚴重程度。

一點也不 非常嚴重

① ② ③ ④ ⑤ ⑥ ⑦ ⑧ ⑨ ⑩

**第五部份：手肘**<br>右側陰影區標示出手肘所指的範圍，您在最近一年內右側陰影區標示出手肘所指的範圍，有沒有疼痛、酸痛、發麻、刺痛或任何不舒服的感覺出現？<br>① 都沒有，本頁不需回答，請跳至下頁。	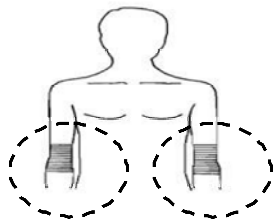
**左手肘** ① 沒有 ，本欄不需填寫。<br>② 有 ，請您務必詳填下列問題。	**右手肘** ① 沒有 ，本欄不需填寫。<br>② 有 ，請您務必詳填下列問題。
1.您的症狀出現的時間為？<br>① 現在 ② 過去一個月<br>③ 過去半年中 ④ 過去一年中<br>2.您的症狀持續多久了？<br>① 1個月 ② 3個月 ③ 6個月<br>④ 1年 ⑤ 3年 ⑥ 3年以上	1.您的症狀出現的時間為？<br>① 現在 ② 過去一個月<br>③ 過去半年中 ④ 過去一年中<br>2.您的症狀持續多久了？<br>① 1個月 ② 3個月 ③ 6個月<br>④ 1年 ⑤ 3年 ⑥ 3年以上

#-;-#

3.您的症狀為(可重複選擇)

① 酸痛 ② 紅腫 ③ 發麻
④ 刺痛 ⑤ 半夜痛醒 ⑥ 肌肉萎縮
⑦ 其他____________________（請說明）

4.您的症狀對您的影響為？

① 完全不影響生活與工作
② 稍微降低工作能力
③ 工作能力明顯降低
④ 曾因此請假休養
⑤ 連生活都受到影響
⑥ 完全不能動作
⑦ 其他____________________（請說明）

5.您的症狀出現頻率為？

① 幾乎每天出現 ② 約一星期一次
③ 約一個月一次 ④ 約半年一次
⑤ 半年以上才出現一次

6.您是否曾尋求治療？

① 未予理會（請跳答第8題） ② 自行處理

③ 中醫 ④ 西醫 ⑤ 中西醫

7.治療方法為(可重複選擇)

① 曾動手術 ② 曾復健 ③ 按摩(推拿)
④ 冷熱敷 ⑤ 吃藥 ⑥ 敷藥
⑦ 其他 ____________________（請說明）

8.您認為造成這些症狀的原因與目前的工作有關嗎？

① 全因工作造成的
② 一部份與工作有關
③ 不清楚
④ 與工作無關，原因是（請說明）

____

9. 請**塗黑**您左手肘範圍的疼痛、酸痛、發麻、刺痛或任何不舒服的感覺出現嚴重程度。

一點也不 非常嚴重

① ② ③ ④ ⑤ ⑥ ⑦ ⑧ ⑨ ⑩

3.您的症狀為(可重複選擇)

① 酸痛 ② 紅腫 ③ 發麻
④ 刺痛 ⑤ 半夜痛醒 ⑥ 肌肉萎縮
⑦ 其他____________________（請說明）

4.您的症狀對您的影響為？

① 完全不影響生活與工作
② 稍微降低工作能力
③ 工作能力明顯降低
④ 曾因此請假休養
⑤ 連生活都受到影響
⑥ 完全不能動作
⑦ 其他____________________（請說明）

5.您的症狀出現頻率為？

① 幾乎每天出現 ② 約一星期一次
③ 約一個月一次 ④ 約半年一次
⑤ 半年以上才出現一次

6.您是否曾尋求治療？

① 未予理會（請跳答第8題） ② 自行處理

③ 中醫 ④ 西醫 ⑤ 中西醫

7.治療方法為(可重複選擇)

① 曾動手術 ② 曾復健 ③ 按摩(推拿)
④ 冷熱敷 ⑤ 吃藥 ⑥ 敷藥
⑦ 其他 ____________________（請說明）

8.您認為造成這些症狀的原因與目前的工作有關嗎？

① 全因工作造成的
② 一部份與工作有關
③ 不清楚
④ 與工作無關，原因是（請說明）

____

9. 請**塗黑**您右手肘範圍的疼痛、酸痛、發麻、刺痛或任何不舒服的感覺出現嚴重程度。

一點也不 非常嚴重

① ② ③ ④ ⑤ ⑥ ⑦ ⑧ ⑨ ⑩

#-<-#

第六部份：手腕

右側陰影區標示出手腕所指的範圍，您在最近一年內右側陰影區標示出手腕所指的範圍，有沒有疼痛、酸痛、發麻、刺痛或任何不舒服的感覺出現？

① 都沒有，本頁不需回答，請跳至下頁。

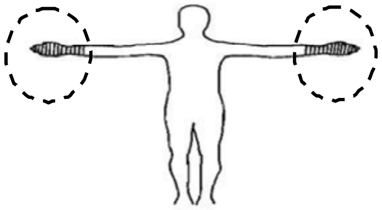

左　腕 ① 沒有　，本欄不需填寫。
② 有　　，請您務必詳填下列問題。

1.您的症狀出現的時間為？

① 現在　② 過去一個月
③ 過去半年中　④ 過去一年中

2.您的症狀持續多久了？

① 1個月　② 3個月　③ 6個月
④ 1年　⑤ 3年　⑥ 3年以上

3.您的症狀為(可重複選擇)

① 酸痛　② 紅腫　③ 發麻
④ 刺痛　⑤ 半夜痛醒　⑥ 肌肉萎縮
⑦ 其他______________（請說明）

4.您的症狀對您的影響為？

① 完全不影響生活與工作
② 稍微降低工作能力
③ 工作能力明顯降低
④ 曾因此請假休養
⑤ 連生活都受到影響
⑥ 完全不能動作
⑦ 其他______________（請說明）

5.您的症狀出現頻率為？

① 幾乎每天出現　② 約一星期一次
③ 約一個月一次　④ 約半年一次
⑤ 半年以上才出現一次

6.您是否曾尋求治療？

① 未予理會（請跳答第8題）② 自行處理
③ 中醫　④ 西醫　⑤ 中西醫

7.治療方法為(可重複選擇)

① 曾動手術　② 曾復健　③ 按摩(推拿)
④ 冷熱敷　⑤ 吃藥　⑥ 敷藥
⑦ 其他 ______________（請說明）

右　腕 ① 沒有　，本欄不需填寫。
② 有　　，請您務必詳填下列問題。

1.您的症狀出現的時間為？

① 現在　② 過去一個月
③ 過去半年中　④ 過去一年中

2.您的症狀持續多久了？

① 1個月　② 3個月　③ 6個月
④ 1年　⑤ 3年　⑥ 3年以上

3.您的症狀為(可重複選擇)

① 酸痛　② 紅腫　③ 發麻
④ 刺痛　⑤ 半夜痛醒　⑥ 肌肉萎縮
⑦ 其他______________（請說明）

4.您的症狀對您的影響為？

① 完全不影響生活與工作
② 稍微降低工作能力
③ 工作能力明顯降低
④ 曾因此請假休養
⑤ 連生活都受到影響
⑥ 完全不能動作
⑦ 其他______________（請說明）

5.您的症狀出現頻率為？

① 幾乎每天出現　② 約一星期一次
③ 約一個月一次　④ 約半年一次
⑤ 半年以上才出現一次

6.您是否曾尋求治療？

① 未予理會（請跳答第8題）② 自行處理
③ 中醫　④ 西醫　⑤ 中西醫

7.治療方法為(可重複選擇)

① 曾動手術　② 曾復健　③ 按摩(推拿)
④ 冷熱敷　⑤ 吃藥　⑥ 敷藥
⑦ 其他 ______________（請說明）

8.您認為造成這些症狀的原因與目前的工作有關嗎？

① 全因工作造成的
② 一部份與工作有關
③ 不清楚
④ 與工作無關，原因是（請說明）______________________

9. 請**塗黑**您左手腕範圍的疼痛、酸痛、發麻、刺痛或任何不舒服的感覺出現嚴重程度。

一點也不 ① ② ③ ④ ⑤ ⑥ ⑦ ⑧ ⑨ ⑩ 非常嚴重

8.您認為造成這些症狀的原因與目前的工作有關嗎？

① 全因工作造成的
② 一部份與工作有關
③ 不清楚
④ 與工作無關，原因是（請說明）______________________

9. 請**塗黑**您右手腕範圍的疼痛、酸痛、發麻、刺痛或任何不舒服的感覺出現嚴重程度。

一點也不 ① ② ③ ④ ⑤ ⑥ ⑦ ⑧ ⑨ ⑩ 非常嚴重

第七部份：臀/大腿

右側陰影區標示出臀/大腿所指的範圍，您在最近一年內右側陰影區標示出臀/大腿所指的範圍，有沒有疼痛、酸痛、發麻、刺痛或任何不舒服的感覺出現？

① 都沒有，本頁不需回答，請跳至下頁。

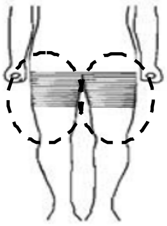

左臀/大腿 ① 沒有 ，本欄不需填寫。
② 有 ，請您務必詳填下列問題。

1.您的症狀出現的時間為？

① 現在 ② 過去一個月
③ 過去半年中 ④ 過去一年中

2.您的症狀持續多久了？

① 1個月 ② 3個月 ③ 6個月
④ 1年 ⑤ 3年 ⑥ 3年以上

3.您的症狀為(可重複選擇)

① 酸痛 ② 紅腫 ③ 發麻
④ 刺痛 ⑤ 半夜痛醒 ⑥ 肌肉萎縮
⑦ 其他______________________（請說明）

4.您的症狀對您的影響為？

① 完全不影響生活與工作
② 稍微降低工作能力
③ 工作能力明顯降低
④ 曾因此請假休養
⑤ 連生活都受到影響
⑥ 完全不能動作
⑦ 其他______________________（請說明）

右臀/大腿 ① 沒有 ，本欄不需填寫。
② 有 ，請您務必詳填下列問題。

1.您的症狀出現的時間為？

① 現在 ② 過去一個月
③ 過去半年中 ④ 過去一年中

2.您的症狀持續多久了？

① 1個月 ② 3個月 ③ 6個月
④ 1年 ⑤ 3年 ⑥ 3年以上

3.您的症狀為(可重複選擇)

① 酸痛 ② 紅腫 ③ 發麻
④ 刺痛 ⑤ 半夜痛醒 ⑥ 肌肉萎縮
⑦ 其他______________________（請說明）

4.您的症狀對您的影響為？

① 完全不影響生活與工作
② 稍微降低工作能力
③ 工作能力明顯降低
④ 曾因此請假休養
⑤ 連生活都受到影響
⑥ 完全不能動作
⑦ 其他______________________（請說明）

5.您的症狀出現頻率為？

① 幾乎每天出現 ② 約一星期一次
③ 約一個月一次 ④ 約半年一次
⑤ 半年以上才出現一次

6.您是否曾尋求治療？

① 未予理會（請跳答第8題） ② 自行處理

③ 中醫 ④ 西醫 ⑤ 中西醫

7.治療方法為(可重複選擇)

① 曾動手術 ② 曾復健 ③ 按摩(推拿)
④ 冷熱敷 ⑤ 吃藥 ⑥ 敷藥
⑦ 其他 ____________________ （請說明）

8.您認為造成這些症狀的原因與目前的工作有關嗎？

① 全因工作造成的
② 一部份與工作有關
③ 不清楚
④ 與工作無關，原因是（請說明）

____

9. 請**塗黑**您左臀/大腿範圍的疼痛、酸痛、發麻、刺痛或任何不舒服的感覺出現嚴重程度。

一點也不 非常嚴重

① ② ③ ④ ⑤ ⑥ ⑦ ⑧ ⑨ ⑩

5.您的症狀出現頻率為？

① 幾乎每天出現 ② 約一星期一次
③ 約一個月一次 ④ 約半年一次
⑤ 半年以上才出現一次

6.您是否曾尋求治療？

① 未予理會（請跳答第8題） ② 自行處理

③ 中醫 ④ 西醫 ⑤ 中西醫

7.治療方法為(可重複選擇)

① 曾動手術 ② 曾復健 ③ 按摩(推拿)
④ 冷熱敷 ⑤ 吃藥 ⑥ 敷藥
⑦ 其他 ____________________ （請說明）

8.您認為造成這些症狀的原因與目前的工作有關嗎？

① 全因工作造成的
② 一部份與工作有關
③ 不清楚
④ 與工作無關，原因是（請說明）

____

9. 請**塗黑**您右臀/大腿範圍的疼痛、酸痛、發麻、刺痛或任何不舒服的感覺出現嚴重程度。

一點也不 非常嚴重

① ② ③ ④ ⑤ ⑥ ⑦ ⑧ ⑨ ⑩

第八部份：膝蓋

右側陰影區標示出膝蓋所指的範圍，您在最近一年內右側陰影區標示出膝蓋所指的範圍，有沒有疼痛、酸痛、發麻、刺痛或任何不舒服的感覺出現？

① 都沒有，本頁不需回答，請跳至下頁。

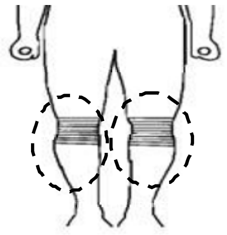

左 膝 蓋 ① 沒有 ，本欄不需填寫。
② 有 ，請您務必詳填下列問題。

1.您的症狀出現的時間為？

① 現在 ② 過去一個月
③ 過去半年中 ④ 過去一年中

右 膝 蓋 ① 沒有 ，本欄不需填寫。
② 有 ，請您務必詳填下列問題。

1.您的症狀出現的時間為？

① 現在 ② 過去一個月
③ 過去半年中 ④ 過去一年中

2.您的症狀持續多久了？

① 1個月 ② 3個月 ③ 6個月
④ 1年 ⑤ 3年 ⑥ 3年以上

3.您的症狀為(可重複選擇)

① 酸痛 ② 紅腫 ③ 發麻
④ 刺痛 ⑤ 半夜痛醒 ⑥ 肌肉萎縮
⑦ 其他________________（請說明）

4.您的症狀對您的影響為？

① 完全不影響生活與工作
② 稍微降低工作能力
③ 工作能力明顯降低
④ 曾因此請假休養
⑤ 連生活都受到影響
⑥ 完全不能動作
⑦ 其他________________（請說明）

5.您的症狀出現頻率為？

① 幾乎每天出現 ② 約一星期一次
③ 約一個月一次 ④ 約半年一次
⑤ 半年以上才出現一次

6.您是否曾尋求治療？

① 未予理會（請跳答第8題） ② 自行處理

③ 中醫 ④ 西醫 ⑤ 中西醫

7.治療方法為(可重複選擇)

① 曾動手術 ② 曾復健 ③ 按摩(推拿)
④ 冷熱敷 ⑤ 吃藥 ⑥ 敷藥
⑦ 其他 ________________（請說明）

8.您認為造成這些症狀的原因與目前的工作有關嗎？

① 全因工作造成的
② 一部份與工作有關
③ 不清楚
④ 與工作無關，原因是（請說明）

____

9. 請**塗黑**您左膝蓋範圍的疼痛、酸痛、發麻、刺痛或任何不舒服的感覺出現嚴重程度。

一點也不 非常嚴重

① ② ③ ④ ⑤ ⑥ ⑦ ⑧ ⑨ ⑩

2.您的症狀持續多久了？

① 1個月 ② 3個月 ③ 6個月
④ 1年 ⑤ 3年 ⑥ 3年以上

3.您的症狀為(可重複選擇)

① 酸痛 ② 紅腫 ③ 發麻
④ 刺痛 ⑤ 半夜痛醒 ⑥ 肌肉萎縮
⑦ 其他________________（請說明）

4.您的症狀對您的影響為？

① 完全不影響生活與工作
② 稍微降低工作能力
③ 工作能力明顯降低
④ 曾因此請假休養
⑤ 連生活都受到影響
⑥ 完全不能動作
⑦ 其他________________（請說明）

5.您的症狀出現頻率為？

① 幾乎每天出現 ② 約一星期一次
③ 約一個月一次 ④ 約半年一次
⑤ 半年以上才出現一次

6.您是否曾尋求治療？

① 未予理會（請跳答第8題） ② 自行處理

③ 中醫 ④ 西醫 ⑤ 中西醫

7.治療方法為(可重複選擇)

① 曾動手術 ② 曾復健 ③ 按摩(推拿)
④ 冷熱敷 ⑤ 吃藥 ⑥ 敷藥
⑦ 其他 ________________（請說明）

8.您認為造成這些症狀的原因與目前的工作有關嗎？

① 全因工作造成的
② 一部份與工作有關
③ 不清楚
④ 與工作無關，原因是（請說明）

____

9. 請**塗黑**您右膝蓋範圍的疼痛、酸痛、發麻、刺痛或任何不舒服的感覺出現嚴重程度。

一點也不 非常嚴重

① ② ③ ④ ⑤ ⑥ ⑦ ⑧ ⑨ ⑩

#-46-#

**第九部份：腳/腳踝**<br>右側陰影區標示出腳/腳踝所指的範圍，您在最近一年內右側陰影區標示出腳/腳踝所指的範圍，有沒有疼痛、酸痛、發麻、刺痛或任何不舒服的感覺出現？<br>① 都沒有，本頁不需回答，請跳至下頁。	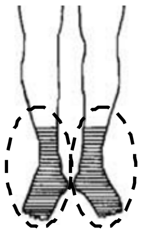
左腳踝/左腳 ① 沒有，本欄不需填寫。<br>② 有，請您務必詳填下列問題。	***右腳踝/右腳*** ① 沒有，本欄不需填寫。<br>② 有，請您務必詳填下列問題。
1.您的症狀出現的時間為？<br>① 現在 ② 過去一個月<br>③ 過去半年中 ④ 過去一年中<br>2.您的症狀持續多久了？<br>① 1個月 ② 3個月 ③ 6個月<br>④ 1年 ⑤ 3年 ⑥ 3年以上<br>3.您的症狀為(可重複選擇)<br>① 酸痛 ② 紅腫 ③ 發麻<br>④ 刺痛 ⑤ 半夜痛醒 ⑥ 肌肉萎縮<br>⑦ 其他________________（請說明）<br>4.您的症狀對您的影響為？<br>① 完全不影響生活與工作<br>② 稍微降低工作能力<br>③ 工作能力明顯降低<br>④ 曾因此請假休養<br>⑤ 連生活都受到影響<br>⑥ 完全不能動作<br>⑦ 其他________________（請說明）<br>5.您的症狀出現頻率為？<br>① 幾乎每天出現 ② 約一星期一次<br>③ 約一個月一次 ④ 約半年一次<br>⑤ 半年以上才出現一次<br>6.您是否曾尋求治療？<br>① 未予理會（請跳答第8題） ② 自行處理<br>③ 中醫 ④ 西醫 ⑤ 中西醫<br>7.治療方法為(可重複選擇)<br>① 曾動手術 ② 曾復健 ③ 按摩(推拿)<br>④ 冷熱敷 ⑤ 吃藥 ⑥ 敷藥<br>⑦ 其他 ________________（請說明）	1.您的症狀出現的時間為？<br>① 現在 ② 過去一個月<br>③ 過去半年中 ④ 過去一年中<br>2.您的症狀持續多久了？<br>① 1個月 ② 3個月 ③ 6個月<br>④ 1年 ⑤ 3年 ⑥ 3年以上<br>3.您的症狀為(可重複選擇)<br>① 酸痛 ② 紅腫 ③ 發麻<br>④ 刺痛 ⑤ 半夜痛醒 ⑥ 肌肉萎縮<br>⑦ 其他________________（請說明）<br>4.您的症狀對您的影響為？<br>① 完全不影響生活與工作<br>② 稍微降低工作能力<br>③ 工作能力明顯降低<br>④ 曾因此請假休養<br>⑤ 連生活都受到影響<br>⑥ 完全不能動作<br>⑦ 其他________________（請說明）<br>5.您的症狀出現頻率為？<br>① 幾乎每天出現 ② 約一星期一次<br>③ 約一個月一次 ④ 約半年一次<br>⑤ 半年以上才出現一次<br>6.您是否曾尋求治療？<br>① 未予理會（請跳答第8題） ② 自行處理<br>③ 中醫 ④ 西醫 ⑤ 中西醫<br>7.治療方法為(可重複選擇)<br>① 曾動手術 ② 曾復健 ③ 按摩(推拿)<br>④ 冷熱敷 ⑤ 吃藥 ⑥ 敷藥<br>⑦ 其他 ________________（請說明）

8.您認為造成這些症狀的原因與目前的工作有關嗎？<br>① 全因工作造成的<br>② 一部份與工作有關<br>③ 不清楚<br>④ 與工作無關，原因是（請說明）<br>____________________<br>9. 請**塗黑**您左腳/腳踝範圍的疼痛、酸痛、發麻、刺痛或任何不舒服的感覺出現嚴重程度。<br>一點也不 ...... 非常嚴重<br>① ② ③ ④ ⑤ ⑥ ⑦ ⑧ ⑨ ⑩	8.您認為造成這些症狀的原因與目前的工作有關嗎？<br>① 全因工作造成的<br>② 一部份與工作有關<br>③ 不清楚<br>④ 與工作無關，原因是（請說明）<br>____________________<br>9. 請**塗黑**您右腳/腳踝範圍的疼痛、酸痛、發麻、刺痛或任何不舒服的感覺出現嚴重程度。<br>一點也不 ...... 非常嚴重<br>① ② ③ ④ ⑤ ⑥ ⑦ ⑧ ⑨ ⑩

參、下肢靜脈曲張不適症狀

右側陰影區標示出下肢靜脈曲張所指的範圍，您在最近一年內右側陰影區標示出下肢靜脈曲張所指的範圍，有沒有疼痛、酸痛、發麻、刺痛或任何不舒服的感覺出現？<br>① 沒有 ，本頁不需回答。<br>② 有 ，請您繼續詳填下列問題。	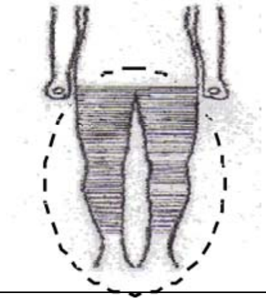

1.您的症狀出現的時間為？

① 現在 ② 過去一個月 ③ 過去半年中 ④ 過去一年中

2.您的症狀持續多久了？

① 1個月 ② 3個月 ③ 6個月 ④ 1年 ⑤ 3年 ⑥ 3年以上

3.您的症狀為(可重複選擇) ？

① 酸痛 ② 紅腫 ③ 發麻 ④ 刺痛 ⑤ 半夜痛醒 ⑥ 肌肉萎縮
⑦ 其他____________________（請說明）

4.您的症狀對您的影響為？

① 完全不影響生活與工作 ② 稍微降低工作能力 ③ 工作能力明顯降低
④ 曾因此請假休養 ⑤ 連生活都受到影響 ⑥ 完全不能動作
⑦ 其他____________________（請說明）

5.您的症狀出現頻率為？

① 幾乎每天出現 ② 約一星期一次 ③ 約一個月一次 ④ 約半年一次
⑤ 半年以上才出現一次

6.您是否曾尋求治療？

① 未予理會（請跳答第8題） ② 自行處理 ③ 中醫 ④ 西醫 ⑤ 中西醫

7.治療方法為(可複選)

① 曾動手術 ② 曾復健 ③ 按摩(推拿) ④ 冷熱敷 ⑤ 吃藥 ⑥ 敷藥
⑦ 其他________________________（請說明）

8.您認為造成這些症狀的原因與目前的工作有關嗎？

① 全因工作造成的
② 一部份與工作有關
③ 不清楚
④ 與工作無關，原因是________________________（請說明）

9. 請**塗黑**您下肢靜脈曲張範圍的疼痛、酸痛、發麻、刺痛或任何不舒服的感覺出現嚴重程度。

一點也不 ... 非常嚴重

① ② ③ ④ ⑤ ⑥ ⑦ ⑧ ⑨ ⑩

【問卷完畢謝謝您】

#-49-#
